# Epigenetic Modifications due to Environment, Ageing, Nutrition, and Endocrine Disrupting Chemicals and Their Effects on the Endocrine System

**DOI:** 10.1155/2020/9251980

**Published:** 2020-07-21

**Authors:** Elizabeth C. Plunk, Sean M. Richards

**Affiliations:** Department of Biological and Environmental Sciences, University of Tennessee, Chattanooga, TN 37403, USA

## Abstract

The epigenome of an individual can be altered by endogenous hormones, environment, age, diet, and exposure to endocrine disrupting chemicals (EDCs), and the effects of these modifications can be seen across generations. Epigenetic modifications to the genome can alter the phenotype of the individual without altering the DNA sequence itself. Epigenetic modifications include DNA methylation, histone modification, and aberrant microRNA (miRNA) expression; they begin during germ cell development and embryogenesis and continue until death. Hormone modulation occurs during the ageing process due to epigenetic modifications. Maternal overnutrition or undernutrition can affect the epigenome of the fetus, and the effects can be seen throughout life. Furthermore, maternal care during the childhood of the offspring can lead to different phenotypes seen in adulthood. Diseases controlled by the endocrine system, such as obesity and diabetes, as well as infertility in females can be associated with epigenetic changes. Not only can these phenotypes be seen in F1, but also some chemical effects can be passed through the germline and have effects transgenerationally, and the phenotypes are seen in F3. The following literature review expands upon these topics and discusses the state of the science related to epigenetic effects of age, diet, and EDCs on the endocrine system.

## 1. Introduction to Epigenetics

Epigenetic reprogramming occurs during germ cell development and embryogenesis, and epigenetic modifications influence the expression of genes, creating phenotypic expression, without changing the genetic sequence [1]. To date, there are three known epigenetic mechanisms of endocrine disruption: DNA methylation, histone modification, and aberrant miRNA expression. These mechanisms have myriad effects on human development, health, and reproduction.

Epigenetic modifications do not alter the gene sequence; however, they can alter gene expression [2]. Epigenetic changes are heritable [3] during cell division [4] and reversible based on environmental signals [5]. Endocrine Disrupting Compounds (EDCs) that methylate DNA can target the cytosine residues located in cytosine-phosphate-guanine (CpG) dinucleotides by adding a methyl group to the 5′ position of the cytosine pyrimidine ring [6]. The importance of methylation at CpG sites and gene expression varies. Gene expression is regulated by cytosine methylation as well as transcription factor binding [7]. It is generally accepted that DNA methylation does not *directly* affect the DNA molecule-with the exception of cytosine methylation at CpG sites. Cytosine methylation is required for embryonic development in mammals and thus is the only epigenetic modification known to directly affect the DNA molecule [5].

Histone modification often occurs concomitantly with DNA methylation and results in short- and long-term alterations in transcription programs [8]. Histones are proteins that pack DNA into nucleosomes which make up chromatin, and modifications in the histone can alter the accessibility of chromatin as well as altering the transcriptional activities in the cell [9]. Gene activation and silencing can be associated with histone modifications [10]. Histone modifications can be responsible for the transduction of hormones such as insulin growth factor I (IGF1) [11].

The third type of epigenetic endocrine disruption is the aberrant expression of microRNAs (miRNAs). miRNAs are closely related to small interfering RNAs (siRNAs) which are involved in DNA methylation and histone modifications [9], and they are composed of 21–24 single stranded nucleotides [12]. miRNAs are noncoding RNA produced from introns/exons that bind to target mRNAs in order to suppress protein translation and posttranscriptional gene expression. Thus, if miRNA expression is amplified or diminished, production of protein or peptide hormones could be disrupted. miRNAs have broad specificity for mRNAs, and more than one miRNA can target mRNAs [13].

## 2. Hormonal Modulation and Endocrine System Plasticity through Epigenetic Mechanisms

The endocrine system is responsible for maintaining homeostasis in the body; therefore, it must be very responsive to environmental alterations [4]. The nutritional environment that the mother has during the last 3 months of pregnancy cues the infant's system as to what the environment will be once out of the womb [14]. The endocrine system will respond to this by altering the metabolic system based on the nutritional environment that the child is suspected to be exposed to [14]. However, this can lead to diseases in child and adulthood if incorrectly adjusted. The child can possibly experience health problems such as cardiovascular disease, diabetes, and obesity if this metabolic adjustment was erroneous [14]. Furthermore, maternal hormonal signals during pregnancy can modify the organizational pathways in the fetus' brain nuclei, which can affect physiological and behavioral responses in the adult offspring [15].

Hormones can affect the phenotype, typically of behavior [16], as well as regulating development, growth, reproduction, metabolism, and immunity [17]. The abundance of hormone receptors themselves can explain the differences between phenotypes among individuals when encountering specific stressors [18]. For example, corticosterone has two receptor types: one that is active when the hormone is present in low concentration (high affinity receptor) and the other that is active when the hormone is in high concentration (the low affinity receptor) [19]. The high affinity receptor is mediating the effects of corticosterone levels daily, and the low affinity receptors are engaged during responses to stress when cortisol is at greatest concentrations, for example [19].

Phenotypic plasticity occurs in response to internal and external environmental cues that lead to the cell changing its behavior [4]. Environmental stressors, both endogenous and exogenous, can partition the genome into active and inactive domains epigenetically, which can drive phenotype plasticity [3]. Critical time periods in epigenetic reprogramming are during gametogenesis and early preimplantation development, and genome-wide demethylation occurs upon fertilization [20]. Furthermore, germ cells and early embryonic cells have been the only cells affected by epigenetic programming on a genome-wide scale [20]. This phenomenon allows for epigenetic traits to be turned from “stable” to “flexible.” Epigenetic programming is important for erasing genomic imprints and epimutations that could be inherited across generations. It also controls transposon silencing [20].

Ong et al. [21] studied the effects of exposing the central amygdala (CeA) to elevated corticosteroids (CORT) on anxiety like behaviors in mice. Researchers inserted pellets of CORT in the CeA in mice. They found that the elevated levels of CORT in the CeA decreased histone acetylation in histone 3 at lysine 9 (H3K9) as well as decreasing glucocorticoid receptors (GR) and increasing corticotropin-releasing factor (CRF) expression. The same researchers also implanted CORT into the dorsal margin of the CeA; GR expression was reduced in the CeA [21]. They also studied the role of histone acetylation in GR and CFR gene expression. In order to do this they treated animals with trichostatin A (TSA) after the CORT implantation. TSA reduces the effects of CORT-induced changes in gene expression. Ong et al. [21] also found that these animals showed greater GR expression in the CeA and showed a reduction in expression of CRF in the CeA. Increasing CORT in the CeA induced H3K9 deacetylation and inhibited histone deacetylases in the CeA and reduced anxiety-like behavior [21].

Ovarian functions such as folliculogenesis, oocyte maturation, ovulation, and luteal function are controlled by microRNA (miRNA) signalling [12]. Aberrant expression of miRNAs can alter endocrine functions. *Dicer* and *Drosha* are essential in the biogenesis of miRNAs [22, 23]. *DGCR8* is a cofactor of *DROSHA* [24]. At embryonic day (E) 6.5, the deletion of *DGCR8* led to the elimination of all miRNA in the embryo and resulted in embryo death [22]. After breeding heterozygously for *Drosha* +, embryos with *Drosha -/-* genotype were much smaller than heterozygous embryos at E6.5, and the embryos deteriorated between E7.5 and E8.5 [22]. A lack of *Drosha* does not cause infertility in aging female mice [25]. In contrast, female fertility in adulthood is not possible without oocyte *Dicer* expression [20]. *Ddx4-Dicer* conditional knockout (cKO) female mice were bred with fertile male mice, and no offspring were produced suggesting *Dicer* cKO leads to infertility [22]. At PND 120 ovaries were evaluated for status of follicles, and *Ddx4-Dicer* cKO ovaries had no developing follicles; interestingly, primordial, primary, secondary, and antral follicles were present at PND 30 and PND 40, suggesting a continuous loss of follicles with age [22].

Wang et al. [24] generated mouse *Dgcr8* knockout embryonic stem (ES) cells in order to assess the role of *Dgcr8* in miRNA processing as well as studying the role of miRNAs in early development. After creating *Dgcr8* knockout ES cells, no mature or intermediate pre-miRNAs were present [24]. Without *Dgcr8,* proper ES cell proliferation and cell-cycle progression cannot occur, and ES cell self-renewal cannot be silenced without miRNAs [24].

Epigenetic mechanisms affect fertility and hormonal responses within an organism and occur due to external and internal stimuli. These modifications and responses can result in anxiety-like behaviors [21], and if the proteins within miRNAs are affected, fertility can be compromised [22].

## 3. Environmental Regulation of Endocrine Systems through Epigenetic Mechanisms

Three genomic targets have been identified as susceptible to environmental epigenetic changes: promoter regions of housekeeping genes, transposable elements that lie adjacent to genes with metastable epialleles, and regulatory elements of imprinted genes [2]. All three targets are rich in CpG dinucleotides, thus making them susceptible. These sequences can be unmethylated, methylated, or differentially methylated between organisms, and some have histone modifications in the same region which determines levels of gene expression [2]. Through environmental stimuli such as exposure to 2,3,7,8-tetrachlorodibenzo-p-dioxin (TCDD), the methylation statuses of *H19* and *Igf2* are altered [26], and other environmental stimuli such as stress lead to the body up- or downregulating enzyme 11*β*-HSD2 which protects the fetus from maternal hormones [27]. The expression of these enzymes can also be altered due to exposure to metals or metalloids [28]. Likewise, maternal care can alter the response to stress later in life of mice by altering the DNA methylation status and chromatin structure [29].

Wu and colleagues [26] directly studied the effects that TCDD has on the methylation status of *H19* and *Igf2* which are growth-related imprinted genes. They exposed mice embryos preimplantation to TCDD and then implanted the embryo in unexposed female mice. With exposure to TCDD the expression level of *H19* decreased, and the expression of *Igf2* tended to decrease but not at a statistically significant value [26]. The 5-methylcytosine concentration in the targeted region of genomic DNA was higher in TCDD-exposed embryos compared to the control. The methylation level of *H19* and *Igf2* in the imprint control region was also higher in TCDD-exposed samples, and this was significantly correlated with lower fetal body weight [26].

Maternal behavior produces stable alterations in DNA methylation and chromatin structure which affects the gene expression in offspring [29]. In a mouse study, adult mice who had high pup licking and grooming (LG) and arched-back nursing (ABN) mothers as infants had a more moderate hypothalamic-pituitary-adrenal (HPA) function, controlled by the neuroendocrine system [30], respond to stress compared to the offspring of low LG-ABN mothers [29]. This outcome is due to the differences in hippocampal glucocorticoid receptor gene (GR) levels and GC negative feedback sensitivity [29]. Using sodium bisulfite mapping, the 5′ and 3′ CpG dinucleotides within the nerve growth factor-inducible protein-A (NGFI-A) binding site were heavily methylated in both the high and low LG-ABN offspring on postnatal day (PND) 1 due to the postnatal wave of new methylation [29]. In the high LG-ABN, the 5′ CpG dinucleotide of the NGFI-A binding site was demethylated by PND 6 [29]. In the offspring of low LG-ABN mothers, the 5′ CpG dinucleotide appears to be methylated always [29].

Even small differences in epigenetic patterns can have an impact on phenotype [31], and the environment can influence these. Monozygous twins begin with the same environment in the womb, and once they are born and throughout life their environment differs from one another. Differences in the genomic distribution of 5-methylcytosine DNA and histone acetylation play a role in gene expression in monozygous twins [32]. These changes can be attributed to both external and internal factors. Nearly one-third of monozygous twins have epigenetic differences in DNA methylation and histone modification. Fraga et al. [32] found that as the twins got older there were more differences in their epigenetic patterns.

Stress during childhood has been linked to reduced glucocorticoid receptors in adults. McGowan et al. [7] found that, in suicide completers who had a history of child abuse, the hippocampal expression of glucocorticoid receptor mRNA and glucocorticoid receptor 1_f_ splice variant was significantly decreased compared to suicide completers without a history of child abuse. Of note, the total glucocorticoid receptor expression is equal to its hippocampal expression. This difference was linked to the increase in methylation of the human glucocorticoid receptor gene (*NR3C1)* and reduced expression of the glucocorticoid receptor mRNA [7].

Glucocorticoids (GCs) are steroid hormones that regulate many functions such as blood pressure and metabolic processes in the body due to their ability to induce many genes' expressions throughout bodily systems [33]. The role of 1*β*-hydroxysteroid dehydrogenase type 2 (11*β*-HSD2) is to protect the fetus from the high levels of maternal GC hormones [28]. 11*β*-HSD2 activity and expression in the placental tissues can be altered by exposure to metals and metalloids [34]. The decrease of expression and activity of 11*β*-HSD2 will result in increased GC exposure and enhanced response to GC in the fetus which will cause GC-induced fetal growth restriction (FGR) [28]. Exposure to cadmium (Cd) through the environment or smoking can decrease 11*β*-HSD2 expression and activity in trophoblast cells in the placenta [28]. In contrast, Mikelson et al. [35] found that there was no correlation between 11*β*-HSD2 and placental concentrations of Cd. Co, Ni, Zn, Fe, and Cu were also evaluated, and there was a positive correlation between the concentration of these metals and the expression of 11*β*-HSD2 only in male placentae, whereas female placentae did not show any correlation between expression of 11*β*-HSD2 and concentration of these metals and metalloids [35].

11*β*-HSD2 activity and expression in the placental tissues can also be altered by maternal stress [27]. An upregulation of 11*β*-HSD2 in the placental tissues can be triggered by maternal acute exposure to stress on GD 20 [27]. In contrast, maternal chronic stress exposure from GD 14 to GD 19 did not alter 11*β*-HSD2 activity; however, it did reduce the ability to upregulate 11*β*-HSD2 activity during acute stress exposure [27].

Environmental factors that affect expression of various genes include exposure to TCDD, maternal behavior to the offspring, stress during childhood, and placental exposure to metals and metalloids. They, respectively, affect the expression of growth imprinting genes *H19* and *Igf2,* GR, *NR3C1,* and 11*β*-HSD2.

## 4. Ageing-Induced Hormone Modulation and Epigenome Modifications

Hormone modulation occurs naturally as we age. GC expression levels increase as we age while the abundance of GC receptors decreases, which can in turn leads to more GC production [33]. In eukaryotic cells, modification of 5-methylcytosine is the most abundant DNA base modification that is responsible for gene repression [36]. During the process of aging, the level of 5-methylcytosine in DNA decreases leading to hypomethylation in most tissues while hypermethylation occurs in promoter regions [37]. As described above, histone modification and DNA methylation often occur concomitantly [8]. Histone modifications can alter the accessibility of chromatin, alter transcriptional activities in the cell [9], and cause transduction of some hormones [11].

The ubiquitin-proteasome system (UPS) deteriorates during the ageing process [38]. Histone deacetylase 1 (HDAC1), DNA cytosine-5-methyltransferase (DNMT1), and chromatin modifiers are regulated by the UPS. Moreover, while ageing, the number of histone chaperones, ASF1A/B, and chromatin assembly factor 1 (CAF1) decreases; therefore the assembly of histones around DNA and histone incorporation into chromatin are also decreased [39].

The role of corticosteroid binding globulins (CBG) is to transport GCs in the blood as well as regulating entry of GCs into the blood. There are no changes in CBG with age [33]. However, the activity of 11*β*-HSD1 enzymes, which convert inactive cortisone into active cortisol [40], increases with age. In contrast, 11*β*-HSD2 activity decreases with age which leads to the increased bioavailability of intracellular GCs. Unregulated GC bioavailability has been implicated in age-related disorders such as metabolic diseases, cognitive decline, and cardiovascular risk [41].

Global hypomethylation can be caused by a decrease in activity of DNMT1, which specifically adds methyl groups to cytosines [42]. Likewise, global hypomethylation can occur due to the inhibition of the activity of DNMT1 by cellular S-adenosylhomocysteine, which increases during aging [42]. Global DNA methylation can decrease due to the reduction of sex hormones during aging [42]. Histone acetylation is also altered during ageing. The irregular histone acetylation is due to the change in activity balance between histone acetyltransferases (HATs) and histone deacetylases (HDACs) that occurs with age. This change can increase the development of age-associated diseases such as insulin resistance [42].

Choi et al. [43] hypothesized that cumulative estrogen exposure across the lifetime may be associated with differential methylation of genes. The reproductive period in a female's life is measured from the age of menarche to the age of menopause, and during this time the woman is exposed to varying activities and types of estrogens [43]. Women with longer reproductive periods have more methylation across the oxidative phosphorylation (OXPHOS) apparatus, which are genes found mainly in autosomes. High levels of methylation in OXPHOS are associated with strokes [43]. Choi et al. [43] also tested their hypothesis by examining the association between reproductive period and DNA methylation in the 95 genes of the 5′ to 3′ UTR [43]. The transcription start site *NDUFS8* showed the most methylation in the OXPHOS apparatus [43]. In the second approach, they [43] used Wilcoxon rank-sum tests to analyze the Hallmark pathways from the Molecular Signatures Database [44] that were the most differentially methylated pathways between organisms in relation to the reproductive period [43]. Ten pathways were differentially methylated; the third most significantly methylated pathway was the Hallmark oxidative phosphorylation pathway [43]. In the Hallmark oxidative phosphorylation pathway, 95 OXPHOS genes overlapped from the first approach, and 68 genes were found to be common; however, 133 genes were unique to the Hallmark pathway [43].

During ageing histone H3.3 becomes more abundant. Histone H3.3 packages the neuronal genome looser than other histones [45]. The looser packaging allows for more histone exchange as well as increased access to transcription machinery [45] and, thus, more histone modification. In baboons, HIRA, a heterochromatin protein and H3.3 specific chaperone found also in humans, increases [46]. Likewise, in embryonic and postnatal development of chicken and mice, H3.3 levels increase in the brain, heart, kidney, liver, and spleen [47].

Epigenetic modifications such as hyper- and hypomethylation and histone modifications can be seen on certain genes through the ageing process.

## 5. Effects of Nutritional Exposure on Endocrine Systems through Epigenetic Route

Bioactive food components can inhibit enzymes that mediate DNA methylation and histone modifications [37]. Nutrients, such as folate, can act as methyl sources that contribute to the production of S-adenosylmethionine (SAM), a methyl donor for methylation reactions [37]. Once SAM donates a methyl group to a methylation reaction, it converts to S-adenosylhomocysteine (SAH), which is further converted to homocysteine [37]. A deficiency in nutrients that donate methyl groups can lead to the lowered availability of SAM and SAH, which decreases the frequency of DNA methylation, due to the decrease of expression of DNMT1 [37]. Epigenetic reductions of DNA methylation in the preovulatory oocyte can have long-term effects on the embryo [48]; furthermore, folic acid supplementation before conception has been linked to the imprinting status of *IGF2* [49].

Insulin-like growth factor 2 (I*GF2*) gene affects growth weight by encoding a fetal and placenta growth factor [50]. During the third trimester maternal concentrations of *IGF2* are inversely related to the mother's body weight and are positively correlated with the placental weight and newborn's height [50]. Lower *IGF2* concentrations during the third trimester on the maternal placental side are correlated with higher *IFG2* differentially methylated region (DMR) 2 [50]. Increased methylation of the *IGF2* DMR is associated with periconceptional folic acid supplementation of the mother [49]. *IGF2* methylation and birth weight are inversely related [49]. 17 months after delivery the biomarkers, SAM, SAH, or SAM/SAH in the mother and child did not differ between periconceptional use of folic acid and no periconceptional use of folic acid [49].

In the weeks leading to conception, sheep were exposed to methyl deficient (MD) diet which included low levels of vitamin B_12_, folate, and amino acid methionine [48]. The MD diet resulted in higher concentrations of homocysteine in ovarian follicular fluid, plasma, and granulosa cell lysates compared to the control diet [48]. The MD offspring showed a greater growth rate until weaning (3 months) compared to the control offspring, and this continued until 22 months of age resulting in heavier MD offspring, with the females being heavier than the males [48]. The body composition did not differ between the MD group and control group until 22 months when the MD males became fatter and had less muscle mass than the control males [48]. MD males were the only group to show insulin resistance, which was independent of adiposity [48]. The higher adiposity and insulin resistance in MD males can be explained by epigenetic modification [48]. Of 1,400 CpG sites analyzed, 57 loci were altered in two or more MD males in comparison to the controls, and 88% of the altered loci were unmethylated or hypomethylated in comparison to the controls [48]. Of the changed loci, 53% were specific to MD males, while only 12% were specific to MD females [48].

Godfrey et al. [51] assessed the methylation status of CpGs in the promoters of candidate genes in umbilical cord tissue collected at birth and the adiposity status of the children at 9 years of age. Of the 31 CpGs that showed hyper- and hypomethylation, 7 had significant associations with the child's adiposity and body fat distribution at 9 years of age [51]. Higher CpG methylation of *RXRA,* which is found among positive regulatory elements of transcription, in the umbilical cord was associated with lower maternal carbohydrate intake, and higher adiposity at 9 years of age [51].

Maternal under- and overnutrition during pregnancy and breast-feeding may affect infant genes that control lipid and carbohydrate metabolism, therefore, inducing alterations in epigenetic routes [52]. Heijmans et al. [53] studied whether periconceptional exposure to famine during the early stages of development is associated with differences in *IGF2* differentially methylated regions (DMR) in adults. Famine exposed individuals were compared to the same-sex siblings [53]. Four of the five CpG sites measured in the *IGF2* DMR showed to be significantly less methylated in individuals exposed to famine periconceptionally compared to their same-sex siblings [53]. Periconceptional exposure to famine was associated with 5.2% lower methylation with no sex-dependent difference [53].

Bogdarina et al. [54] provided pregnant rats with either 20% protein rat chow, which accounts for normal protein consumption (control), or 8% protein rat chow which accounts for maternal low protein (MLP) [54]. This diet was given from pregnancy to weaning which occurred at 3 weeks of age of offspring. Liver, lung, kidney, brain, heart, and adrenal gland tissues were harvested and analyzed at week 1 and week 12. In MLP female offspring at 1 and 12 weeks there was an increase in expression of AT_1a_ angiotensin receptor in the kidney. In male and female MLP offspring, there was an increase in AT_1b_ angiotensin receptor in the adrenal gland, which is associated with the development of hypertension [54]. However, a decrease in expression was found in the AT_2_ receptor in MLP offspring at 1 and 12 weeks. In the liver, angiotensinogen and AT_1a_ receptors showed increased expression at week 1, but the expression normalized by week 12. The methylation status of the 17 CpG sites in the AT_1a_ promoter was evaluated, and no difference was found between the methylation frequency of this region of control offspring and the MLP offspring [54].

Nutritional intake by the mother can lead to hyper- or hypomethylation of genes important to fetal development, and the effects of these epigenetic modifications can be seen after infancy.

## 6. Endocrine Disruptors and Endocrine Responses through Epigenetic Routes

Endocrine disrupting chemicals (EDCs) have a variety of mechanisms. They are structural similarity to hormones, and their mechanisms include altering normal hormone concentrations, inhibiting or stimulating the production and metabolism of hormones, or changing hormones' movement through the body [55]. These actions can result in the production of adverse developmental, reproductive, neurological, and immune effects in humans [17]. EDCs can cause effects at low doses in a tissue-specific manner, and the age at which a person is exposed to EDCs can determine their effects [55]. Prenatal exposure can lead to reproductive pathologies [55], neurodevelopmental delays in children [17], and metabolic and hormonal disorders later in life by altering normal cellular and tissue development and function during developmental programming [55].

Androgens and estrogens, steroid hormones, are involved in normal growth and development of human secondary sex organs [56]. Three types of estrogen receptors that EDCs can interact with are nuclear estrogen receptors (ER*α* and ER*β*) which are essential in transcription regulation [57], membrane bound estrogen receptors, and estrogen G protein-coupled receptor (GPR30) [55]. Xenoestrogens can function by utilizing membrane bound receptors and second messenger pathways, an indirect pathway [58], as well as disrupting normal signalling pathways [59]. Indirect pathways can be activated at low xenoestrogen concentrations and can lead to nongenomic effects on gene expression [58]. The nongenomic effects can be perpetuated by continuous ligand stimulation and sending signals downstream, which can cause genomic effects once the signals are in a position to control the activation state of transcription factors [59].

Anderson et al. [57] analyzed the relationship between nuclear receptors and histone methylation modifiers in embryonic testis tissue in mice. Nuclear receptor *Rarb* and methyltransferase *Suv39h1* are both present in similar expression concentration in efferent ducts, epididymis, and vas deferens in embryonic tissue; likewise, nuclear receptor *Nr1h2* and methyltransferase *Suv420h2* show a similar relationship [57]. A strong correlated expression is present in the embryonic tissue from efferent ducts, epididymis, and vas deferens between the nuclear receptor *Rarb* and methyltransferase *Suv39h1* as well as between the nuclear receptor *Nr1h2* and the methyltransferase *Suv420h2* [57].

Histone modification can act as a gatekeeper mechanism by promoting or preventing the promoter access to liganded-nuclear receptors [60]. The gatekeeper theory may explain the differing phenotypes displayed when the same genes with diverse functional roles are exposed to EDCs [57]. The gatekeeper concept was studied in genes in testis by exposing rats from gestational day (GD) 6 to PND 92 to low and high doses of myclobutanil, propiconazole, and triadimefon. All are endocrine disrupting fungicides. Anderson et al. [57] determined the gatekeeper set to be the methyltransferases: *Ehmt1, Ehmt2, Prdm2,* and *Setdb1.* The expression of the receptor genes *Ar* (androgen receptor gene) and *Esr2* (estrogen receptor gene) was studied in relation to the expression of the identified gatekeeper methyltransferases. In the samples exposed to high doses of triadimefon, the gatekeeper set had highly correlated expression with the receptors. In the 22 phenotypic genes studies, nine did not show coexpression with either *Ar* or *Esr2.* Three had coexpression approaching but not reaching significance with *Esr2.* Ten showed statistically significant coexpression with *Esr2, Ar,* or both. In contrast, only two samples exposed to low dose of propiconazole had two gatekeeper genes showing high coexpression with each other, and none showed coexpression with a nuclear receptor, which is important in transcription regulation. These results support the concept of the gatekeeper mechanism where histone methylation modifiers work in unison with nuclear receptors to mediate transcriptional change in target genes [57].

Brominated flame retardants (BFRs) are *in vitro* and *in vivo* endocrine disruptors [61] that structurally resemble polychlorinated bisphenyls (PCB) [62]. There are many BFR congeners that affect thyroid hormones, and polybrominated diphenyl ether (PBDE) congeners affect spermatogenesis at doses as low as 60 ug/kg/bw [61]. BFRs can also bind to estrogen receptors [62]. Kamstra et al. [63] studied the effects of the BFR 2,2′,4,4′-tetrabrominated diphenyl ether (BDE-47) on 3T3-L1 adipocyte differentiation on *in vitro* adipocytes. After 8 days of exposure to BDE-47, they [63] found demethylation of CpG regions and increased gene expression of *Ppary2,* peroxisome proliferator activated receptor, and gamma 2. Kamstra and colleagues found significant demethylation of the 3 CpG regions between base pairs −337 and −192. The reason for increased gene expression of *Ppary2* is due to decreased methylation in the *Ppary2* promoter [63].

Exposure to endocrine disruptors such as fungicides and BFRs can affect the expression of various receptors due to differential methylation and histone modifications.

## 7. Epigenetic Transgenerational Inheritance of Endocrine Diseases Promoted by Ageing, Diet, and Environmental Endocrine Disruptors

Sex-steroid hormones establish methylation status during critical developmental periods [64], and epigenetic modifications begin as early as germ cell development and embryogenesis [1]. EDCs can act on sex-steroid hormone receptors, so during critical developmental periods if the fetus is exposed to EDCs, remethylation could occur within the germ cells, and these effects can be observed in subsequent generations [64]. Transgenerational effects, involving the transmission of epigenetic changes in the germline, occur when effects from the endocrine disruptor are observed without direct exposure, or in F3 [65, 66]. In contrast, if passed down by the paternal lineage, the epigenetic phenotype becomes transgenerational once expressed in the F2 generation [66]. During adult life, if the F0 generation is exposed to endocrine disruptors, preconceptionally the F1 generation is being directly exposed [65]. Multigenerational effects are when any effects of the endocrine disruptor are observed in the F1 and F2 generations [65]. Some examples of environmental EDCs that can cause transgenerational effects are bisphenol-A (BPA), Di(2-ethylhexyl) phthalate (DEHP), and vinclozolin [65] (Table 1).

Vinclozolin is a fungicide known for its antiandrogenic endocrine disruption action [75]. Vinclozolin was the first EDC shown to display transgenerational inheritance [76]. Nilsson et al. [77] transiently exposed pregnant rats to vinclozolin, DDT, or control (DMSO) during GD 8–14. There was no increase of the ovarian diseases polycystic ovarian syndrome and primary ovarian insufficiency, in F1 and F2 generations from exposure to DDT and vinclozolin in mice, but there was an increase in the two ovarian diseases in F3 generation [77]. After ancestral exposure to DDT and vinclozolin, changes in DNA methylation in the F3 generation were present in the areas of the genome with relatively low CpG density [77]. Similarly, maternal exposure to diethylstilbestrol (DES) at a dose of 10 *μ*g/kg/maternal body weight results in increased proliferative lesions (PPL) in the oviduct in F2 mice [67].

Inawaka et al. [68] examined whether the antiandrogens vinclozolin, procymidone, or flutamide caused transgenerational effects of DNA methylation in male rats. These researchers exposed maternal mice from GD 8 to GD 15 to 100 mg/kg/day vinclozolin, 100 mg/kg/day procymidone, or 10 mg/kg/day flutamide. DNA methylation analysis on 210 base pairs including 7 CpG sites in the *lysophospholipase* gene on F1 male pups occurred on PND 6. Exposed F1 males not sacrificed on PND 6 were bred with untreated-females, and subsequent DNA methylation analysis occurred on F2 generation on PND 6. DNA methylation status was comparable to the control, and no transgenerational effects were observed due to the DNA methylation caused by antiandrogens exposure to the F1 males [68].

Bisphenol-A (BPA) is a synthetic compound used in plastics and resins. Exposure to 5 mg/kg BPA prenatally disrupts the number of ER *α*-cells in brain regions that play a role in reproductive function in female mice [65]. Likewise, exposure to the same dose perinatally increases Meg3, an epigenetic modifier, expression in the female hypothalamus in F3 generation females. Increased concentrations of BPA and subsequent elevated expression of Meg3 are associated with precocious puberty in women and laboratory mice [65]. For example, in mice, ancestral exposure to BPA at 0.5, 20, and 50 *μ*g/kg/day proved to cause dysregulated gene expression of ovarian apoptotic factors, oxidative stress factors, and autophagy factors [69]. The exposure dose of 5 mg/kg in mice is estimated to be what is present in human maternal blood, 0.3–18.9 ng/mL, making this dose environmentally relevant [78–80].

Furthermore, in a study examining the transgenerational effects of an environmental dose of 20 *μ*g/L of BPA in female zebrafish, alterations in genes involved in female reproduction at a transcriptional level were found [70]. Variation in transcript abundance for the genes *esr, star, lhcgr,* and *fshr* was observed through F3 [70]. The transcript abundance of *amh,* a gene involved in gonadal differentiation, was reduced up to F3 due to hypermethylation of its promoter regions as well as alterations in H3K4me3/H3K27me3 [70].

In male rats, the lowest effective doses of BPA to reduce male fertility are 1.2 and 2.4 *μ*g/kg bw [71]. Exposure to 1.2 *μ*g and 2.4 *μ*g BPA perinatally led to significantly reduced sperm count and sperm motility in F1, F2, and F3 males [71]. A decrease in ER*β* was observed in all generations of both exposure groups, while in both F1 generations ER*α* expression was increased [71]. *Ar* expression was decreased in F1, F2, and F3 males in the 1.2 *μ*g group, while in the 2.4 *μ*g group a decrease was only seen in F3 [71]. The altered phenotype caused by perinatal exposure to BPA is seen transgenerationally; therefore, BPA exposure perinatally possibly caused reprogramming in the epigenome of the germ cells [81].

DEHP is a plasticizer found in numerous consumer products and is associated with transgenerational epigenetic effects in the ovaries of mice. Brehm et al. [72] studied the transgenerational effects of DEHP exposure at doses 20 *μ*g/kg/d, 200 *μ*g/kg/d, 500 mg/kg/d, and 750 mg/kg/d. Beginning on GD 11, the pregnant females were dosed with a solution of DEHP orally. Prenatal exposure to DEHP did not affect body weight or liver weight in the F1 and F2 generations [72]. In the F2 and F3 generations uterine weight was not affected by DEHP exposure. In the F1 generation ovarian weight decreased at a dose of 20 *μ*g/kg/d, and uterine weight increased at 500 and 750 mg/kg/d doses. In the F3 generation, transgenerational effects observed were decreased body weight (200 *μ*g/kg/d), decreased ovarian weight (20 and 200 *μ*g/kg/d and 500 mg/kg/d), and decreased liver weight (20 and 200 *μ*g/kg/d and 500 and 750 mg/kg/d) [72]. In the F1 generation, Brehm et al. [72] found that 750 mg/kg/d DEHP increased the time spent in proestrus and metestrus/diestrus while decreasing the time spent in estrus. There were no observable effects in the F2 generation. In F3 a dose of 20 *μ*g/kg/d decreased the time spent in proestrus; likewise, exposure to 200 *μ*g/kg/d of DEHP decreased the time spent in proestrus and increased the time spent in estrus and metestrus/diestrus [72]. Exposure to 500 mg/kg/d decreased the time spent in proestrus and estrus while increasing the time spent in metestrus/diestrus. Finally, 750 mg/kg/d increased the time spent in metestrus/diestrus [72]. Up to F3 reduced ovarian follicular reserve and oocyte and blastocyst developmental competence can be seen [82]. DEHP also affected the expression of genes responsible for trophoblast differentiation and implantation until F4 in mice [82]. The expression of *Lif-R* was upregulated in F2 and F3 generations with a dose of 0.05 and 5 mg DEHP/kg/day, and, in F4 *Lif-R*, it was upregulated due to dose of 0.5 mg/kg/day [82].

Male mice exposed to 150 mg/kg DEHP perinatally led to lighter seminal vesicles transgenerationally which suggests decreased testosterone levels [83]. In exposures of 200 mg/kg, the male mice showed behavior differences in comparison to the controls, which can be due to differing corticosterone levels [83].

Brehm et al. [72] also studied the effects of DEHP exposure on folliculogenesis which is possibly due to DNA methylation. In the F1 generation, they found that a dose of 750 mg/kg/d decreased the number of primordial follicles, and, at a dose of 20 *μ*g/kg/d, the number of preantral follicles was decreased. In the F2 generation, the number of primordial follicles was increased at a dose of 500 mg/kg/day and the number of primary follicles increased at 200 *μ*g/kg/d [72]. One transgenerational effect was observed at 200 *μ*g/kg/d and the effect was the increase of the number of primordial follicles [72]. In the F1 generations, 500 mg/kg/d of DEPH increased the levels of estradiol and decreased the levels of testosterone, and 750 mg/kg/d increased the levels of estradiol in mice [72]. In the F2 generation, 20 *μ*g/kg/d decreased testosterone and 200 ug/kg/d decreased progesterone [72]. The transgenerational effects were seen in 20 *μ*g/kg/d when levels of estradiol increased and levels of testosterone decreased [72]. Testosterone decrease was also seen at a dose of 500 mg/kg/d [72].

Exposure to phthalate diethylhexyl phthalate in midgestation causes puberty delay in F1 and F3 generation males [73]. Other phenotypes in the F3 generation include lower sperm counts, testicular germ cell function, and increased incidence of abnormal seminiferous tubules [73].

Manikkam et al. [74] studied the transgenerational effects of TCDD. F0 generation female rats were exposed to TCDD from fetal days 8 to 14; then they were bred to produce F1 [74]. Only F1 and F3 generation adult rats were evaluated [74]. In F3 females, the body, ovarian, and uterine weights showed no change, while the kidney weight was reduced [74]. In F3 males, testis, epididymis, and prostate weights did not change, while kidney weight was reduced [74]. Serum testosterone concentrations were increased in F3 males, and serum estradiol concentration in F3 females during proestrus-estrus phase or diestrus phase showed no change [74]. These results led to the conclusion that F3 males experienced endocrine alterations while F3 females did not [74]. F1 and F3 females show an increase in primordial follicle loss and polycystic ovarian disease, and in F3 male sperm 50 differentially DNA methylated regions in promoters were found [74]. Overall, 50 statistically significant differentially DNA methylated regions in promoters between F3 males sperm epigenome were found [74].

Fungicides, BPA, DEHP, and TCDD affect reproduction transgenerationally in both males and females due to epigenetic modifications.

## 8. Conclusion

Epigenetic modifications due to exposure to different nutrients pre- and postnatally, EDCs, maternal behavior, and ageing can lead to various endocrine phenotypes. The endocrine system is susceptible to changes in the environment due to its role in maintaining homeostasis. *In utero*, the endocrine system predicts the environment the fetus will be living in, and the epigenetic reprogramming, if wrong, can lead to diseases such as cardiovascular disease, obesity, and diabetes. Hyper- and hypomethylation while ageing is responsible for the alterations in concentrations of hormones, hormone receptors, and DNMTs. Maternal exposure to stress, metals, or metalloids can alter the expression of 11*β*-HSD2 in the placenta leaving the fetus unprotected from maternal GCs. EDCs such as fungicides, BPA, DEHP, and TCDD have transgenerational effects, seen in F3 (Table 1). In contrast, DES and antiandrogens only have multigenerational effects, seen in F2. Further research is needed to explore whether the concentrations in which humans are exposed to various EDCs cause epigenetic effects. The field of epigenetics and the effects on the endocrine system is growing, and more research is needed to see if the alterations in gene expression is solely due to epigenetic modifications or if other mechanisms are at play.

## Figures and Tables

**Table 1:** EDCs and their effects as discussed in Section 7.

EDC	Animal model	Sex	Dose	Effect	Generation	Reference
DES	Mouse	Female	10 *μ*g/kg/maternal bw	Increased PPL of oviduct	F2	[67]
Vinclozolin	Rat	Male	100 mg/kg/day	No effect on methylation	N/A	[68]
Procymidone	Rat	Male	100 mg/kg/day	No effect on methylation	N/A	
Flutamide	Rat	Male	10 mg/kg/day	No effect on methylation	N/A	
BPA	Mice	Female	5mg/kg	Increased Meg3	F3	[65]
	Mice	Female	0.5, 20, 50 *μ*g/kg/day	Dysregulated gene expression of ovarian apoptotic factors, oxidative stress factors, autophagy factors	N/A	[69]
	Zebrafish	Female	20 *μ*g/L	Variations in transcript abundance of genes *Iesr, star, lhcgr, fshr, amh*	Up to F3	[70]
	Rats	Male	1.2 *μ*g and 2.4 *μ*g	Reduced sperm count and motility, decreased ER*β* expression	Up to F3	[71]
				Increased ER*α* expression	F1	
			1.2 *μ*g	Decreased *Ar* expression	Up to F3	
			2.4 *μ*g	Decreased *Ar* expression	Only F3	
DEHP	Mice	Female	20 *μ*g/kg/d	Ovarian weight decrease	F1	[72]
			500 and 750 mg/kg/d	Uterine weight increase	F1	
			200 *μ*g/kg/d	Decreased body weight	F3	
			20 and 200 *μ*g/kg/d, 500 mg/kg/d	Decreased ovarian weight	F3	
			20 and 200 *μ*g/kg/d and 500 and 750 mg/kg/d	Decreased liver weight	F3	
			750 mg/kg/d	Increased time in proestrus and metestrus/diestrus	F1	
			20 and 200 *μ*g/kg/d	Decreased time in proestrus	F3	
			200 *μ*g/kg/d	Increased time in estrus and metestrus/diestrus	F3	
			500 mg/kg/d and 750 mg/kg/d	Increased time in metestrus/diestrus	F3	
			500 mg/kg/d	Decreased time in proestrus and estrus	F3	
			750 mg/kg/d	Decreased number of primordial follicles	F1	
			20 *μ*g/kg/d	Decreased number of preantral follicles	F1	
			200 *μ*g/kg/d 500 mg/kg/d	Increased number of primordial follicles	F2	
			200 *μ*g/kg/d	Increased number of primary follicles	F2	
			500 mg/kg/d	Increased levels of estradiol and decreased levels of testosterone	F1	
			750 mg/kg/d	Increased levels of estradiol	F1	
			20 *μ*g/kg/d	Decreased testosterone and increased levels of estradiol	F2	
			200 *μ*g/kg/d	Decreased progesterone	F2	
			500 mg/kg/d	Decreased testosterone	F2	
		Male	150 mg/kg	Lighter seminal vesicles	N/A	[73]
			200 mg/kg	Behavioral differences	N/A	
TCDD	Rats	Female	N/A	Reduced kidney weight	F3	[74]
				Increased primordial follicle loss and polycystic ovarian disease	F1 & F3	
		Male	N/A	Reduced kidney weight and increase in serum testosterone concentrations	F3	

## Abbreviations

ABN: Arched-back nursing
BFRs: Brominated flame retardants
BPA: Bisphenol-A
CAF1: Chromatin assembly factor 1
CBG: Corticosteroid binding globulins
CpG: Cytosine-phosphate-guanine
cKO: Conditional knockout
DEHP: Di(2-ethylhexyl) phthalate
DNMT1: DNA cytosine-5-methyltransferase
DES: Diethylstilbestrol
EDC: Endocrine disrupting chemical
ES: Embryonic stem
GCs: Glucocorticoids
GR: Glucocorticoid receptor gene
HDAC1: Histone deacetylase 1
HPA: hypothalamic-pituitary-adrenal
IGF2: Insulin-like growth factor 2
LG: Licking and grooming
MD: Methyl deficient
MLP: Maternal low protein
miRNA: MicroRNA
PBDE: Polybrominated diphenyl ether
PCB: Polychlorinated bisphenyls
PND: Postnatal day
SAH: S-Adenosylhomocysteine
Sam: S-Adenosylmethionine
TCDD: 2,3,7,8-Tetrachlorodibenzo-p-dioxin
UPS: Ubiquitin-proteasome system.
